# Black and Latinx Primary Caregiver Considerations for Developing and Implementing a Machine Learning–Based Model for Detecting Child Abuse and Neglect With Implications for Racial Bias Reduction: Qualitative Interview Study With Primary Caregivers

**DOI:** 10.2196/40194

**Published:** 2023-01-31

**Authors:** Aviv Y Landau, Ashley Blanchard, Nia Atkins, Stephanie Salazar, Kenrick Cato, Desmond U Patton, Maxim Topaz

**Affiliations:** 1 School of Social Policy & Practice University of Pennsylvania Philadelphia, PA United States; 2 New York Presbyterian Morgan Stanley Children’s Hospital Columbia University Irving Medical Center New York, NY United States; 3 Columbia College Columbia University New York, NY United States; 4 Columbia School of Social Work Columbia University New York, NY United States; 5 University of Pennsylvania School of Nursing University of Pennsylvania Phildelphia, PA United States; 6 Childrens Hospital of Philadelphia University of Pennsylvania Phildelphia, PA United States; 7 Annenberg School for Communication University of Pennsylvania Phildelphia, PA United States; 8 Department of Child and Adolescent Psychiatry and Behavioral Sciences University of Pennsylvania Phildelphia, PA United States; 9 Columbia University School of Nursing Columbia University New York, NY United States; 10 Columbia University Data Science Institute Columbia University New York, NY United States

**Keywords:** child abuse and neglect, pediatric emergency departments, machine learning–based risk models, electronic health records, primary caregivers, child, abuse, neglect, model, epidemic, development, implementation, community, machine learning

## Abstract

**Background:**

Child abuse and neglect, once viewed as a social problem, is now an epidemic. Moreover, health providers agree that existing stereotypes may link racial and social class issues to child abuse. The broad adoption of electronic health records (EHRs) in clinical settings offers a new avenue for addressing this epidemic. To reduce racial bias and improve the development, implementation, and outcomes of machine learning (ML)–based models that use EHR data, it is crucial to involve marginalized members of the community in the process.

**Objective:**

This study elicited Black and Latinx primary caregivers' viewpoints regarding child abuse and neglect while living in underserved communities to highlight considerations for designing an ML-based model for detecting child abuse and neglect in emergency departments (EDs) with implications for racial bias reduction and future interventions.

**Methods:**

We conducted a qualitative study using in-depth interviews with 20 Black and Latinx primary caregivers whose children were cared for at a single pediatric tertiary-care ED to gain insights about child abuse and neglect and their experiences with health providers.

**Results:**

Three central themes were developed in the coding process: (1) primary caregivers’ perspectives on the definition of child abuse and neglect, (2) primary caregivers’ experiences with health providers and medical documentation, and (3) primary caregivers’ perceptions of child protective services.

**Conclusions:**

Our findings highlight essential considerations from primary caregivers for developing an ML-based model for detecting child abuse and neglect in ED settings. This includes how to define child abuse and neglect from a primary caregiver lens. Miscommunication between patients and health providers can potentially lead to a misdiagnosis, and therefore, have a negative impact on medical documentation. Additionally, the outcome and application of the ML-based models for detecting abuse and neglect may cause additional harm than expected to the community. Further research is needed to validate these findings and integrate them into creating an ML-based model.

## Introduction

### Background

Child abuse and neglect is defined as any action (physical, sexual, or emotional) by a caregiver that results in harm, potential harm, or threat of harm to a minor [1]. Child abuse and neglect is significant public health concerns in the United States and worldwide. In 2019, over 4.4 million referrals concerning some form of child abuse and neglect were made to child protective services across the United States [2]. Detecting child abuse and neglect in clinical practice is challenging as there is no “gold standard” for identification [3]. Additionally, there is a level of consensus among health providers that existing stereotypes may tie race and social class to child abuse [4]. For example, Black and Latinx patients are twice as likely as White parents to be evaluated and reported for suspected abusive head trauma [5]. Moreover, Black children born in the United States have an estimated 53% chance of being investigated by child protective services by the time they reach adulthood. White children, in comparison, have a 23.4% chance [6]. These disproportionate rates of investigations of Black families by child protective services have raised questions about reimagining social and child support systems [7], and redefining child abuse and neglect detection and interventions based on the sociocultural contexts of marginalized communities [8].

Across-the-board adoption of electronic health records (EHRs) in health settings has supported the development of clinical decision support systems for identifying potential child abuse and neglect, thus offering innovative avenues for detection and intervention [9,10]. To improve the development, implementation, and outcomes of machine learning (ML)–based models that use EHR data, it is essential to involve stakeholders and community members in the process. This involvement allows for a better understanding of how and when medical data are collected [11].

This exploratory research is part of a broader study that aims to develop an ML-based model to detect child abuse and neglect in emergency departments (EDs) with implications for racial bias reduction and future interventions. In our past research, health providers (social workers, nurses, and physicians) who provided important considerations for generating a phenotype for child abuse and neglect using EHR data were interviewed. First, there are information-related challenges that include a lack of proper previous visit history due to limited information exchanges and scattered documentation within EHRs. Second, there are differences in the documentation styles in how child abuse and neglect is described by health providers. For example, social workers provide richer context and in-depth descriptions of social and family history and plan of care in their clinical notes. At the same time, nurses focus on signs and symptoms, and physicians tend to provide detailed information regarding injuries related to potential abuse and neglect. And third, medical documentation could potentially help identify health discrepancies in the quality of care concerning child abuse and neglect [12]. Furthermore, we developed a set of ethical recommendations concerning the development and evaluation of ML-based models for detecting child abuse and neglect, which included the importance of involving community members and stakeholders in the process [13]. In compliance with our previous research and ethical guidelines, there is a need for additional research to gain perspectives of primary caregivers, who are likely to be affected by such ML systems, on child abuse and neglect.

This exploratory research strives to design ethical and inclusive ML-based models for detecting potential child abuse and neglect and reduce racial bias in EDs. We recognize the importance of mandated reporting and are aware that health care providers play a key role in detecting child abuse and neglect. For this reason, it is imperative to ensure that ML developers consider primary caregivers’ thoughts around child abuse and neglect prior to developing a tool that quantitatively identifies child abuse and neglect. As a first step, we interviewed Black and Latinx primary caregivers to gain insight into their perspectives on the definitions of and ways to report child abuse and neglect and their experiences in health care settings.

### Objective

The general goal of this work is to develop an ML-based model for detecting child abuse and neglect in ED settings using EHR documentation with implications for reducing racial bias. This qualitative study aimed to elicit Black and Latinx primary caregivers’ perspectives about child abuse and neglect while living in underserved communities to highlight implications for developing and implementing an ML-based model for detecting child abuse and neglect.

## Methods

### Study Design

We conducted a qualitative study through in-depth interviews with 20 Black and Latinx primary caregivers whose children were cared for at a single pediatric tertiary-care ED. The participants provided insights about child abuse and neglect and their experience with health providers to enhance the development of an ML-based model to detect child abuse and neglect and reduce racial bias in ED settings.

### Participants

To design an equitable ML-based model that aims to reduce inequity in health systems and understand the impact of racial bias in child abuse and neglect reporting, it is critical to partner with, listen to, and collect meaningful data from marginalized communities [14]. All participants in this study were Black or Latinx identified as primary caregivers of a child who presented to a single pediatric ED in the Northeast region of the United States. We recruited primary caregivers based on the racial and ethnic composition of the marginalized population served by the ED. Primary caregivers were recruited through a purposive sampling strategy. Purposive sampling methods included ED pediatricians formally asking primary caregivers to participate in the research after receiving medical attention for their children. Inclusion criteria encompassed (1) primary caregivers had a child taken care of in the pediatric ED, (2) primary caregivers identified themselves as Latinx or Black, and (3) primary caregivers were proficient in English.

The participants include single primary caregivers (n=12), divorced primary caregivers (n=4), and primary caregivers who are married (n=4). The average age of the primary caregiver was 35.5 (range 25-47, SD 6.34) years. All participants sought medical care for their children at the ED. Primary caregivers who were recruited were not evaluated for child abuse and neglect. Primary caregivers had up to 4 children (mean 2.05 children, SD 0.94). The primary caregivers interviewed identified themselves as Latinx (n=15), Black (n=4), and mixed race (n=1). All participants recruited were mothers who accompanied their children to the ED. The recruitment of mothers was not intentional but resulted from COVID-19 policies allowing only 1 caregiver to accompany a child in the ED.

### Procedure

In-depth one-on-one interviews were conducted between October 2020 and April 2021. The number of interviewees was determined by theoretical saturation; interviews took place until no new themes emerged during the continuing review of transcripts [15]. SS and NA conducted interviews. SS and NA are research assistants whom AYL and DUP trained in qualitative research. SS and NA are Black and Latinx and have focused their academic or professional careers on promoting social justice. All 20 interviews were carried out in English by NA and SS who understand both English and Spanish to better help with translation when needed for cultural nuances. The interviews were conducted on the phone due to COVID-19 restrictions and health protocols, thus enabling flexible time opportunities for proceeding with interviews. Interviews were between 30 minutes and 1 hour. All interviews were audio-recorded and transcribed by SS and NA before analysis. Primary caregivers received a US $100 gift card to participate in this research.

A semistructured interview guide was designed to gain insights on primary caregivers' perceptions of child abuse and neglect, experiences with health care systems and providers, and perceptions of how and when child abuse and neglect is reported to child protective services. We used the following questions to guide the qualitative interviews: (1) How has becoming a parent impacted how you think about your definition of child abuse or neglect? (2) Tell us about your experience encountering medical staff in regards to your child's health care? (3) Can you describe a time when someone you know was accused of child neglect or abuse?

### Data Analysis

We applied a thematic analysis approach to identify and generate key themes and categories [16,17]. This analysis comprises 3 stages. At stage 1, 3 members of the research team engaged in open coding to facilitate a process of crystallization by reading the interviews multiple times in order to become familiar with the content and enhance a reflexive approach, and an understanding and interpretation of the data so that coding can begin. This method provides more in-depth and complex insight [18]. Next, using Dedoose [19], a qualitative software, the researchers' SS, NA, and AL first coded 2 identical transcripts individually to explore various meanings and patterns within the data, identify initial areas of controversy to debate within the extended group, refine the codebook, and develop a code scheme. In this phase, we created codes such as “thoughts of child abuse and neglect.”

In stage 2, we explored the remaining transcriptions while simultaneously comparing codes and grouping and sorting key codes into initial categories [16,17]. Categories were then compared, aligned, and assembled into key themes that provide meaning and underline overarching patterns in the data. Categories were added, removed, and changed throughout the analysis based on the continuous familiarization of content. For example, based on our initial code, “thoughts of child abuse and neglect,” we zoomed in on how one’s upbringing and life experience can help shape thoughts concerning child abuse and neglect. We then developed the codes “Discipline examples” and “Cultural upbringing” to label content that emphasizes how primary caregivers have different parenting styles and how they describe their upbringing. Second, these codes were compiled into categories such as “One’s upbringing influences the Definition of child abuse.” Third, the researchers compiled this category under the central theme “Primary caregivers definitions for child abuse and neglect,” which highlights a critical understanding of how primary caregivers define and perceive child abuse and neglect phenomena.

In stage 3, the final themes were discussed, refined, and carefully completed by the research team collaboratively. We divided existing categories to specify the challenges primary caregivers from marginalized communities encounter when encountering health providers. For example, a category called “information shared with and by health providers” was divided into 2 new categories: “Language barriers between health providers and the primary caregivers” and “Primary caregivers perception concerning medical documentation and questionnaires.”

### Ethical Considerations

This study received ethical approval from the institutional review board at Columbia University (IRB-AAAS9840). Prior to the interviews, all participants provided informed consent. The study's verbal and written aims were presented to the primary caregivers, including the right to refuse to participate or to end the interview. In addition, participants were informed of the sensitive nature of this research, which includes topics such as race, child abuse and neglect, and health disparities. The research assistants were instructed to involve the research team and provide participants with the phone number of the principal investigator who is a trained social worker, if any emotional concerns arose. All the participants' names were changed to maintain and protect their anonymity.

## Results

### Overview

Three central themes were generated from the analysis: (1) primary caregivers’ definition of child abuse and neglect: although there is a consensus on child neglect, defining child abuse from a primary caregiver's perspective is challenging due to the multiple manifestations of abuse, which are judged by the severity of physical and emotional violence toward the child and considered within the social and cultural context; (2) primary caregivers’ experiences with health providers and medical documentation: the existence of communication barriers between patient and health provider due to language barriers and ambiguous medical documentation; and (3) primary caregivers’ perceptions of child protective services: child protective services are considered a potential threat for families in marginalized communities.

### Theme 1: Primary Caregivers’ Definition of Child Abuse and Neglect

This theme includes similarities and differences between primary caregivers’ perceptions about child abuse and neglect (Textbox 1). The majority (90%) of primary caregivers had similar understandings of child neglect. For example, primary caregivers described child neglect as a lack of food, hygiene, appropriate clothing, shelter, education, and emotional support (quotes Q1-Q3).

Similar to child neglect, primary caregivers also had a consensus on how they define child abuse. Most primary caregivers (n=15, 75%) described child abuse not only as physical abuse but also as emotional, verbal, and sexual abuse (Q4), which can range from inappropriate verbal communication to child molestation (Q5-Q6). In addition, when describing child abuse, some primary caregivers referred to different levels of physical contact as the primary indicator of abusive behavior. One primary caregiver, a single 41-year-old mother of 2, pointed out that minimal physical contact results in a negligible amount of harm and thus is not considered as abuse (Q7). Two other primary caregivers described child abuse as beating a child excessively (Q8) and emphasized that the presence of physical signs, such as scars or broken bones, is what differentiates an abusive act from a nonabusive act (Q9).

**Primary caregivers’ definitions for child abuse and neglect.**

**Primary caregivers’ consensus for defining child neglect**

Quote 1 [Q1]: *Child neglect is where basically you’re not taking care of your child. Your child’s hungry, dirty, just needs to be paid attention, and you’re just not doing that. The parent is not nurturing the child, bathing the child, or even playing with the child.* [Taylor]

Q2: *When you don't take care of your child, when you just don't feed them, don’t clothe them, don't give them the emotional support that they need.* [Leticia]

Q3: *Neglect would be parents not providing appropriate food, shelter, livable conditions; Like lack of food, dirty clothing, absences from school.* [Samantha]

**Primary caregivers’ definitions of child abuse**

Q4: *Child abuse is a lot of things. It’s emotional, physical, sexual….* [Alyssa]

Q5: *Abuse can be either physical or verbal. I can imagine screaming, cursing, foul language at the child, and spanking a child.* [Yerely]

Q6: *I define child abuse by hitting children in an inappropriate way that you're not supposed to hit. Child abuse can be also molesting children, doing things that you're not supposed to do or touching them or doing things in an inappropriate way of doing it.* [Nadia]

**Child abuse is defined by different severity of physical contact**

Q7: *I think that if you hit them a little bit, it’s not going to kill them.* [Leticia]

Q8*: For me, it’s if you’re beating the child excessively.* [Taylor]

Q9*: I described that as when you make physical contact with your child, but not only when you hit them like nicely or small, but when you cause some problems, like you break a bone or scar them or something like that.* [Dorothy]

**One’s upbringing influences the Definition of child abuse**

Q10: *It’s when we were talking about punishment or how to discipline a child. For example, some parents when their child doesn't do what they want, they just abuse them physically. Hitting them with something like a belt or a shoe. I’m from the Dominican Republic and back in the time, I used to see a lot of abuse.* [Adriana]

Q11: *Many different ways of child abuse are mental, physical, emotional; with children it’s just tricky to know because they’re children, and sometimes the way you were raised kind of hinders your parenting because you don't know how to break away from those cycles.* [Liora]

Q12: *I don't believe in physically disciplining a child at a young age, I did get disciplined but like I said, that don't mean I would do it with mines.* [Tania]

Finally, primary caregivers relayed how perceptions around child abuse can be influenced by one’s upbringing and sociocultural context. For example, Adriana described how growing up in the Dominican Republic, she witnessed how discipline is taught through acts of force and abuse (Q10). These childhood experiences continue to impact parenting styles, and even as these behavioral cycles are hard to break (Q11), Tania, a mother of 2, emphasized how similar upbringing experiences can also result in avoiding abusive behavior (Q12).

### Theme 2: Primary Caregivers’ Experiences With Health Providers and Medical Documentation

Primary caregivers might have multiple day-to-day encounters with health care providers in the community, ranging from visits with neighborhood pediatricians to receiving medical attention at a hospital’s ED. This theme highlights the different challenges primary caregivers may have when encountering health providers (Textbox 2). A majority of primary caregivers (N=11: Latinx=7, Black=4) recalled experiencing some communication challenges with health providers in regards to language barriers and medical terminology.

Miscommunication due to language barriers is a central challenge between health providers and non–English-speaking patients and families. Spanish-speaking primary caregivers described their experience with health providers who do not speak Spanish and how that impacted the quality of care. Dorothy, a 28-year-old mother of one, shared her feelings that health providers may potentially not pay attention to patients who do not speak English (Q13). Vanessa strengthened the above and further explained the importance of providing interpreters, who are beneficial for patients in communicating about their health status (Q14). Even if the health provider has some background with the patients' language, this background remains insufficient and can cause discomfort for patients receiving medical treatment (Q15). However, it is essential to note that when a health provider is fluent in other languages such as Spanish, this fluency enhances the quality of care and signals a sense of understanding and respect for the patient (Q16).

**Primary caregivers’ experience with health providers and documentation.**

**Language barriers between health providers and the primary caregivers**

Q13: *Sometimes [the health providers] don’t know Spanish, or mostly they don't know [the patients’] language, and [the patients] feel like [the providers] don’t pay attention to them because they don’t understand.* [Dorothy]

Q14: *I think the biggest thing in the Spanish community…was if you spoke Spanish and that was your primary language, that was a big issue, because there wasn’t a lot of interpreters or it was too much of a hassle for them to find an interpreter and during that time or even now I feel a lot of people in the Spanish community have a hard time talking to doctors or letting them know what’s going on when they can’t speak the language of the doctor.* [Vanessa]

Q15: *When it comes to translation, [Mother] felt like “oh they didn't get somebody to translate just because the doctor speaks a little bit of Spanish.” “[the doctor] tried to figure out what I was having, but I felt uncomfortable. I wasn't getting the answer that I wanted.”* [Rocio]

Q16: *I have nothing bad to say, the doctors are so nice and respectful and because I speak mostly Spanish, sometimes I don’t understand, and so they try to explain things to me in a way that I understand. Now there are more people who speak Spanish there and all of them speak Spanish really well and now they ask “oh what do you prefer, Spanish or English?” But before, it wasn’t like that. They could speak Spanish, but not that much.* [Alondra]

**Primary caregivers’ perception concerning medical documentation and questionnaires**

Q17: *I always tell people—tell me in layman's terms. I didn't go to medical school. I don't understand the things that you’re doing, but at the same time, I want to be helpful not just to the nurses and doctors but to my child as well, when they are asking me “why are they doing this or why do I need this for?”* [Fatima]

Q18: *One thing they did do that I did notice after reading his discharge papers is they put both the medical term for something and then the more casual, kid-friendly term in parentheses next to it and they also put the reason as to why he is taking it, so he’s taking this because of inflammation, whatever, etc. I really appreciate it because people ask me “oh what kind of medication is he taking?” And so, I tell them, “this is the medication he’s taking and this is the reason why.”* [Alyssa]

Q19: *If I’m being honest with myself, if I don’t feel ok with answering the questions. It's not because I’m afraid of answering the questions. I'm afraid of being perceived—as a mother you know your kids look put together, but then you get these questionnaires and they speak to you at a level where you're like “should I admit or should I not?” like you're afraid like “what happened to my kids?,” “how are they going to see me?” These are questions that sometimes people have a hard time answering.* [Liora]

Q20: *At this time I never knew about caseworkers or social workers, so this lady called me to her office and was asking me questions and I felt comfortable. So, with me being comfortable I'm opening myself up, and I'm being blunt, and I'm letting that person in, but then it goes sideways on talking about Maria (the caregiver’s other daughter who was not the original subject of conversation) and how Maria is coming up in the household and that shut me down and I felt so uncomfortable and, I felt like, “am I doing something?”* [Tania]

During medical treatment, besides the traditional medical procedures, primary caregivers often need to read medical documentation and go through medical evaluations and questionnaires concerning their children's health and their own parenting styles. Primary caregivers highlighted that they prefer health providers to relay medical information and documentation in nonprofessional language to better understand their children's clinical condition and treatment. Receiving understandable medical documentation is rewarding and appreciated by primary caregivers because it allows them to be more involved in their child's care (Q17-Q18). Finally, study participants shared their concerns and fears regarding health questionnaires that evaluate their child's health and care. Liora, a 28-year-old mother of 2, explained how she is afraid when asked health-related questions because she worries that her answers will negatively impact how she is perceived as a mother (Q19). Similarly, Tania shared how she felt uncomfortable talking with a hospital social worker about her other child who did not need medical attention—“*I'm letting that person in, but then it goes sideways on talking about Maria”* (Q20).

### Theme 3: Primary Caregivers’ Perceptions of Child Protective Services

Primary caregivers suggested that reports to child protective services may occur due to false accusations by neighbors, children, and mandatory reporters such as counselors and social workers (Textbox 3). In total, 5 (25%) study participants shared their thoughts around false accusations. For example, a primary caregiver suggested that false accusations concerning a child's safety occur “millions of times” due to disputes within the neighborhood (Q21). Another primary caregiver provided an additional aspect of how children may have false perceptions concerning their parents' involvement. Leticia described how her son shared with a counselor that she is not present, and afterward, she needed to explain that she is taking care of her other child, who is in the hospital. Due to her experience, Leticia also acknowledged the need to reform child abuse and neglect reporting laws (Q22). Along a similar vein, Kena suggested that when parents are falsely accused of abuse and neglect, they are the ones who need to advocate and prove that no wrongdoing happened (Q23).

**Primary caregivers provide their insights on child protective services.**

**Primary caregivers share their experiences and knowledge about false accusations concerning child abuse and neglect**

Q21: *Oh falsely accused? Millions of times. In the projects, there’s a lot of that, that happens here. People get angry or upset at something and they think because you know—I think that the access to reporting child abuse is way too easy. I think that you know you call child protective services and then it’s—It has to be viable evidence for you to call this person and say ‘I heard yelling’ and they automatically think it's a child in danger but it's usually just the opposite.* [Liora]

Q22: *My own son one day, he went to the guidance counselor and I had to go explain to her that my daughter has medical problems and he has never been left alone. He’s left with my mom and my brother. Of course, I stay in the hospital with my daughter but he’s not by himself. He’s never been by himself. So, it depends on how the kid’s perception also might be totally wrong and the adult doesn’t know. So, I think they have to seriously reform how these laws are made.* [Leticia]

Q23: *So I was falsely accused of child abuse with ACS. And again, I had to advocate for myself I had to show them that what they were saying was not accurate.* [Kena]

## Discussion

### Principal Findings

This qualitative study provides several insights from primary caregivers concerning definitions of child abuse and neglect, interactions between health providers and primary caregivers, and perceptions of child protective services. These perceptions are critical to consider when designing an equitable and ethical ML-based module for detecting child abuse and neglect in EDs [14].

In this study, the majority of primary caregivers had similar perceptions of child neglect—parents not providing their children with adequate nutrition, hygiene, clothing, protection, education, and emotional support. These findings are supported by previous research that indicated that the public consensually defined child neglect as unmet physical and emotional needs due to a lack of appropriate parenting [20].

Primary caregivers also described how child abuse occurs through physical, sexual, and emotionally abusive behaviors. There is a broad understanding of what child abuse entails; primary caregivers shared that the physical indicators and the severity of physical contact need further consideration when evaluating child abuse. Past research has demonstrated similar findings. For example, Price et al [21] described a need to develop parental education to help primary caregivers understand the difference between child abuse and appropriate disciplinary actions.

Our findings indicate that the primary caregivers' upbringings and sociocultural backgrounds influence their discipline styles, perspectives of child abuse and neglect, and ability to engage with other forms of parenting. These findings support the importance of contextually understanding the influence of primary caregivers' culture and community to expand knowledge of the etiology and prevention of child abuse and neglect [8]. With that said, our study included only caregivers who identify as Black and Latinx; thus, the findings do not suggest that due to cultural factors, Black and Latinx caregivers are predisposed to severely disciplining their children, or are more inclined to do so than caregivers of other races and ethnicities.

In this study, participants shared the importance of bridging language barriers between health providers and primary caregivers to improve the quality of care. Specifically, language barriers cause patients to feel neglected and limit their chances to articulate health concerns and learn more about the medical diagnosis. Thus, these barriers foster a sense of burden, stigma, discrimination, and frustration among patients [22,23], potentially impacting how health providers detect child abuse and neglect.

US law prohibits health providers from blocking information and requires health providers to share EHR data with their patients [24]. Sharing medical documentation with patients is far more beneficial than the perceived risks (privacy breaches, confusion, and lack of medical knowledge) [25]. Participants expressed that providing nonprofessional and understandable medical information and documentation is rewarding and appreciated. Easy-to-read medical documentation enhances primary caregivers' sense of participation and control concerning their children's health and care. Moreover, patients who have access to their medical documentation can verify potential errors, improve their understanding of child’s health problems, and potentially have lower levels of stress [26]. Therefore, open medical documentation and EHR designed to reduce racial and social bias can potentially assist families when advocating for their children [27].

In addition, primary caregivers also highlighted their concerns regarding answering and providing in-depth and sensitive information via medical evaluations and questionnaires. Participants felt afraid and uncomfortable and were concerned about how health providers may perceive their parental qualities.

Finally, developing ML based-models to detect child abuse and neglect without considering the potential outcomes and primary caregivers’ perceptions of child protective services can potentially perpetuate harmful and traumatizing experiences, especially for Black and Latinx primary caregivers. Primary caregivers in this study illustrated how false accusations concerning child abuse and neglect occasionally occur without the possibility to control and advocate against those false accusations. Recent studies strengthen our findings and show that child protective services mainly concentrate their involvement in low-income and marginalized communities and invest significant time and resources investigating parents reported for child abuse and neglect [28,29], thus disproportionately removing Black and Latinx children from their homes [6,7].

### Primary Caregivers Implications for Developing and Implementing an ML-Based Model to Help Identify Child Abuse and Neglect

Our findings have several implications for developing and implementing an ML-based model to help identify potential child abuse and neglect in ED settings. First, although there are clinical and legal definitions for child abuse and neglect, members of marginalized communities may have different perspectives and life experiences that inform their definitions of abuse and neglect. When developing ML-based models to help identify child abuse and neglect, it is critical to have open discussions between primary caregivers, social workers, and other health professionals to understand diverse perspectives regarding children's health and safety. These discussions will contribute to a better universal definition for child abuse and neglect, thus altering labeling practices and determining child abuse and neglect classifications. Second, when incorporating unstructured and structured medical data in developing ML-based models, researchers should consider language barriers between nonnative English-speaking patients and health providers. Communication challenges may impact medical documentation, data curation, and inclusivity in data practices. Miscommunication could lead to misinterpretation of symptoms and can potentially result in a misidentification that is reflected in the medical documentation.

Third, we recommend improving the readability and redesigning medical documentation by involving diverse voices that can provide feedback and recommendations for designing medical documentation. Lengthy medical questionnaires and ambiguous medical jargon can result in frustration and a lack of trust between health providers and patients, thus impacting the identification and potential reporting of abuse and neglect.

Fourth, members of marginalized communities and primary caregivers may perceive child protective services as threatening. Our findings demonstrate that child protective services may be weaponized to hurt families by way of false accusations. Moreover, scholars such as Dorothy Roberts recommend abolishing child protective services as they harm Black and Latinx Families [29]. Therefore, it is essential to weigh the consequences of developing and implementing ML-based models that help identify potential child abuse. ML developers should engage with community members to gain insight into the dynamics between families and child protective services. Moreover, ML developers should work closely with primary caregivers throughout the development and implementation processes to reduce bias and better validate the ML-based model.

Finally, ML developers of ML-based models that identify child abuse and neglect should test for racial and ethnic bias in the classifications and during the model development process [30]. Mitigating these biases is crucial considering that unwarranted interactions with child protective services may have long-lasting negative effects on caregivers, their families, and their community.

### Limitations

This research has several significant limitations. First, we interviewed only primary caregivers of patients from 1 ED of a large urban hospital. In future research, it is essential to interview primary caregivers from multiple locations to gain deeper insight and perspective. Second, all primary caregivers were mothers with different living situations. Future research should include interviewing children, fathers, and other caregivers with different relationship and marital statuses. Third, we interviewed only Black and Latinx primary caregivers. Future studies should gather insights from other marginalized populations, such as immigrants and members of the LGBTQ community. Fourth, our research raised several sensitive questions around abuse and neglect, mandatory reporting of racial bias, and child protective services. Further studies should explore these issues through a quantitative lens as well.

### Implications for Further Research

Additional research is needed in three areas: (1) exploring additional methodologies for including community members in designing ML-based models for detecting child abuse and neglect, (2) addressing sociocultural aspects in defining and detecting child abuse and neglect, and (3) developing an antiracist lens for using EHR data.

### Conclusions

To our knowledge, this is the first qualitative study to gain insights from primary caregivers for developing an ML-based model for detecting child abuse and neglect. Our findings present several challenges that ML developers need to consider when designing and developing detection tools for child abuse and neglect. This includes how to define child abuse and neglect from a primary caregiver lens. Miscommunication between patient and health provider can potentially lead to a misidentification, and therefore have an impact on medical documentation. Furthermore, the outcome and implementation of the ML-based models to detect child abuse and neglect may cause additional harm to the community than expected. Further studies are needed to validate these findings and incorporate them into the ML-based model for detecting child abuse and neglect.

## Abbreviations

ED: emergency department
EHR: electronic health record
ML: machine learning

## Data Availability

The data underlying this article cannot be shared publicly to maintain the privacy of individuals that participated in the study. The data will be shared upon reasonable request to the corresponding author.
